# “Preparation Is Key”: Parents’ and Nurses’ Perceptions of Combined Parent-Delivered Pain Management in Neonatal Care

**DOI:** 10.3390/children11070781

**Published:** 2024-06-27

**Authors:** Martina Carlsen Misic, Emma Olsson, Ylva Thernström Blomqvist, Alexandra Ullsten

**Affiliations:** 1 Department of Pediatrics, Faculty of Medicine and Health, Örebro University, 70182 Örebro, Sweden; 2 Faculty of Medicine and Health, School of Health Sciences, Örebro University, 70182 Örebro, Sweden; 3 Neonatal Intensive Care Unit, University Hospital, 75185 Uppsala, Sweden; 4 Department of Women’s and Children’s Health, Uppsala University, 75237 Uppsala, Sweden; 5 Centre for Clinical Research and Education, Region Värmland, 65182 Karlstad, Sweden

**Keywords:** infant, neonate, pain management, parent, parent-delivered intervention

## Abstract

Background: There is a knowledge-to-practice gap regarding parent-delivered pain management, and few studies have investigated parents’ and nurses’ participation in and acceptance of combined parent-delivered pain-alleviating interventions such as skin-to-skin contact (SSC), breastfeeding, and parental musical presence. This study investigated parents’ and nurses’ perceptions of and reflections on experiencing combined parent-delivered pain management. Methods: This qualitative study applies a collaborative participatory action research design using ethnographic data collection methods such as focus groups, video observations, and video-stimulated recall interviews with parents and nurses. Results: The results concern three main categories, i.e., preparation, participation, and closeness, as well as various sub-categories. Preparations were central to enabling combined parent-delivered pain management. Participation was facilitated by parental musical presence, in which parents shifted their attention toward their infant. Closeness and presence during neonatal care helped parents become active during their infant’s painful procedures. Parental lullaby singing created a calm and trusting atmosphere and after the procedure, both parents and nurses felt that they had successfully supported the infant through a potentially painful procedure. Conclusions: Mental and practical preparation is central to implementing combined parent-delivered pain management. When parents and nurses explored the interventions, they found the methods feasible, promoting self-efficacy and confidence in both parents and nurses.

## 1. Introduction

Parents find infant pain a distressing aspect of their infant’s hospitalization in the neonatal care unit, and they are eager to participate in comforting their infant during painful procedures [1,2]. Parents are a valuable but often overlooked and underutilized resource in neonatal pain care, and healthcare staff attitudes and beliefs play a central role in successful parental involvement [1].

It is well known that infants admitted to neonatal care are exposed to 7 to 17 painful procedures every day [3]. Far from every infant will receive adequate pain management during these procedures [4]. Non-pharmacological methods, such as skin-to-skin contact (SSC), are recommended as the first choice in procedural pain management in infants because they are considered safe and without side effects [5]. SSC refers to the infant being cared for on their parent’s bare chest wearing only a diaper and possibly a cap and has several positive physiological and behavioral effects on preterm and newborn infants, both healthy and ill, and on parents in terms of less stress and improved self-efficacy [6]. Several studies have demonstrated SSC to have pain-relieving effects [7,8]. Breastfeeding is another non-pharmacological intervention that has demonstrated effectiveness equal to, or greater than, that of sweet solutions in reducing behavioral and physiological responses to procedural pain in full-term infants [9,10,11]. To be effective, breastfeeding should start at least two minutes before the procedure and the infants need to suck effectively before, during, and after the painful procedure [11].

The soothing, comforting, and emotion-regulating properties of lullabies are well known cross-culturally and historically [12]. Live singing has been shown to enhance parents’ intuitive communicative abilities [13], and parents who are encouraged to sing with their infant gradually gain confidence in communicating vocally with their infant within a short time [14]. In procedural pain management, recorded lullabies and recorded maternal voices, both singing and talking, have been shown to have some positive effects on infants’ behavioral and physiological pain indicators, resulting in, for example, increased oxygen levels, lowered heart rate, and reduced pain scores [15,16]. A small randomized controlled study found that mothers’ live infant-directed speech and singing during a heel stick procedure modulated the preterm infants’ pain indicators and increased the oxytocin levels in both mothers and infants [17]. However, there remains a paucity of research on combined multisensorial parent-delivered interventions, including their musical and relational aspects [1].

Parent-delivered pain management is defined as interventions delivered by parents during painful procedures in the care of newborn infants in hospital settings [18]. Optimal pain management for hospitalized infants during procedures involves parent-delivered pain-alleviating interventions [19]. Combining several non-pharmacological methods seems to be more effective than using a single method [20]. Research has shown that the combination of SSC and breastfeeding is more effective in reducing infants’ responses to pain than either intervention alone [9,11]. However, a knowledge-to-practice gap regarding parent-delivered pain management remains, and few studies have investigated parents’ and nurses’ participation in and acceptance of combined parent-delivered pain-alleviating interventions such as SSC, breastfeeding, and parental musical presence [1]. To our knowledge, this is the first study exploring healthcare professionals’ and parents’ views and reflections on experiencing combined parent-delivered methods in which live parental lullaby singing is included.

The aim of this study was to investigate parents’ and nurses’ perceptions of and reflections on experiencing combined parent-delivered pain management.

## 2. Materials and Methods

### 2.1. Design

This study used an explorative inductive qualitative design inspired by participatory action research (PAR). In PAR, researchers and participants work together to understand a problem and ameliorate it [21,22]. In this study, parents, nurses, and the researchers jointly explored combined parent-delivered pain management. The researchers were active participants in the PAR design. MCM and AU performed all data collection to maintain consistency throughout the process. MCM is an experienced neonatal nurse and AU is an experienced neonatal music therapist and researcher.

### 2.2. Participants and Recruitment

The data were collected in three neonatal care units with level 2/3 care [23] in Sweden. By means of convenience sampling, we recruited nine parents whose infants were currently being cared for in NICUs; we also recruited two veteran parents with earlier experience of neonatal care, three registered nurses with a specialist degree, and one assistant nurse, for a total of fifteen participants (Table 1). Two registered nurses and the assistant nurse participated twice in data collection. In this paper, the registered nurses and assistant nurse are referred to simply as nurses.

Recruitment of families to the study was challenging: one family dropped out due to transfer to another unit, and two families left the units before the focus group took place, which extended the time needed for data collection. Three additional families were invited but declined to participate.

### 2.3. Data Collection

The data were collected between September 2021 and May 2023. By triangulating data collection methods, our aim was to develop a comprehensive understanding of the phenomenon of combined parent-delivered pain management. The data were collected using the following ethnographic methods: focus group discussions with instructional and informative elements, video recordings and participant observations (interpreted in a detailed description, see Appendix A), field notes, and video-stimulated recall interviews [24].

Due to the ongoing COVID-19 pandemic when the study started, it was not possible for the participants to meet in person and the focus groups took place on a secure online platform. During data collection, the restrictions for COVID-19 were released but the focus groups were still performed online to keep consistency and to facilitate attendance for the participants. Due to the logistics of performing the focus groups, not all invited participants were able to participate, resulting in a smaller sample than planned. A total of five focus groups (Table 2) were performed, lasting 48–71 min.

**Figure 1:**
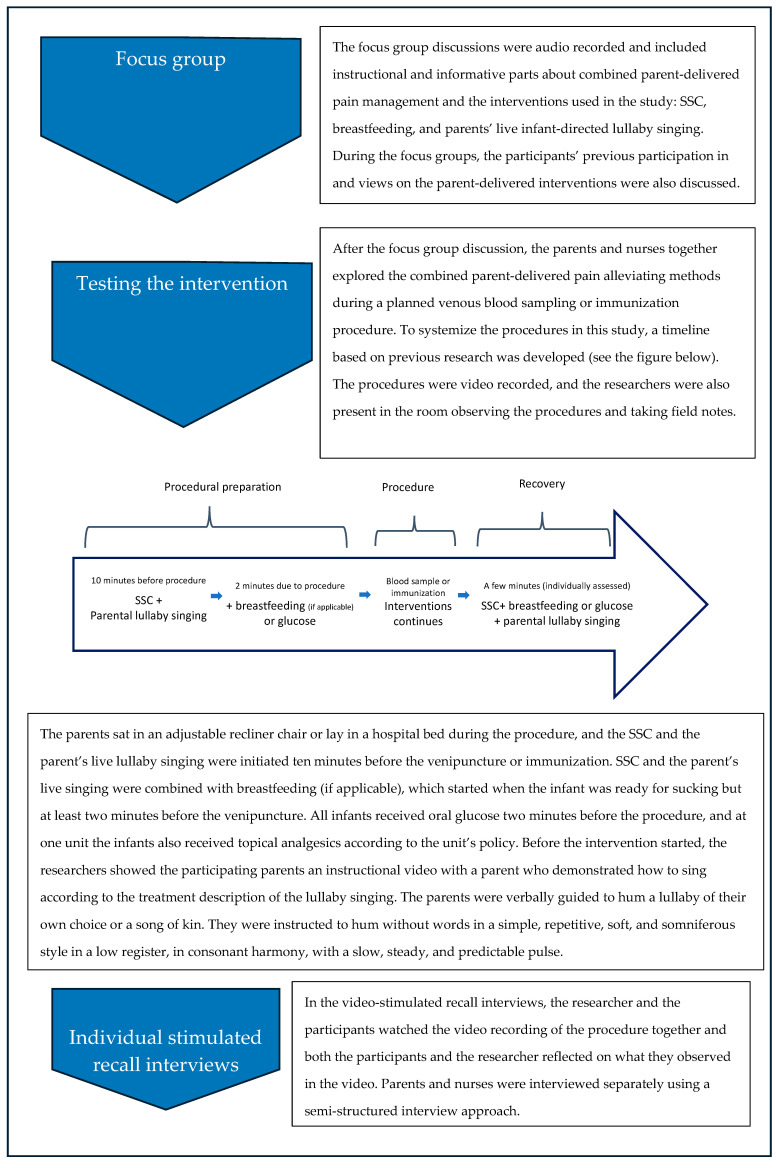
An overview of the data collection process.

To prepare the last step of data collection, the researchers used microanalysis [25] to hand code the videos second by second, observing parents’, nurses’, and infants’ activities and potential critical incidents during the venipunctures or immunizations. The researchers then used their field notes and microanalysis to inform the video-stimulated recall interviews, which lasted 20–52 min. In-depth descriptions (Appendix A) summarized the context of the procedures in detail, such as the environment, gestures, and sequences of events [26]. An overview of the data collection process is presented in Figure 1.

### 2.4. Data Analysis

The focus group discussions and individual video-stimulated recall interviews were analyzed using Malterud’s systematic text condensation [27] (Table 3).

There was an iterative process of moving back and forth among the described steps. As recommended by Malterud, a preliminary analysis of the interviews was conducted. On two occasions during data collection, MCM, AU, and EO met and jointly read the material to identify meaning units and preliminary themes. The preliminary analysis led to minor changes in the interview guides. When all of the data were collected and all analysis steps were performed, the final categories and sub-categories were identified. All authors performed steps one and two (see Table 3); MCM performed the final steps of the analysis and then met with the whole research group to discuss and confirm the final content and meaning of the data.

### 2.5. Ethical Considerations

The present study was approved by the Swedish Ethical Review Authority (Dnr 2020-01562). The participants received both written and verbal information about the study. Written informed consent was collected from all participants. The present study upheld the ethical conduct of neonatal pain trials [28] by providing pain management for all involved infants.

## 3. Results

An ethnographic detailed description is presented in Appendix A, in which the first author (MCM) chose one of the procedures and contextualized the phenomenon of combined parent-delivered pain management. From the emic perspective of the participating observer, the first author described and interpreted the observed activities, behaviors, and interactions of the participants, including the infant, within the context of testing combined parent-delivered pain management.

**Figure 2:**
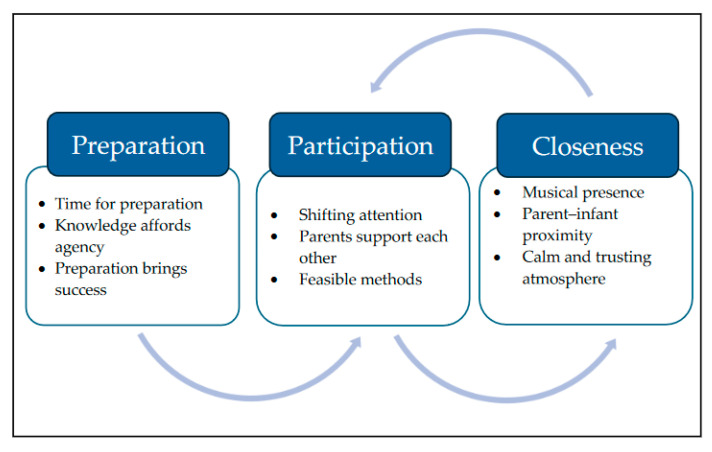
An overview of the main categories and sub-categories.

The results of the analysis of the video-stimulated recall interviews consisted of three main categories: preparation, participation, and closeness, including nine sub-categories. There was no hierarchy among the categories, but the preparation category was the most extensive. The analysis identified how the three categories enabled one another (Figure 2). Before a painful procedure, preparation is the first step and forms the foundation for participation and closeness during the procedure. There is an iterative flow in which participation and closeness facilitate each other.

### 3.1. Preparation

Both parents and nurses considered preparation crucial for combined parent-delivered pain management. This category involved long-term preparations, starting directly after the birth of the infant with early parental responsibilities and information from the staff. It also consisted of preparations undertaken before the studied procedure. All parents identified the need for sufficient time to calm themselves and mentally prepare for the procedure. The preparation category also consisted of adaptations of the environment to facilitate the combined parent-delivered pain management.

#### 3.1.1. Time for Preparation

This study included a predetermined time of ten minutes of preparation. Both nurses and parents discovered the importance of this preparation time, which was essential for the parents and infant to become relaxed before the procedure:

He usually calms down quite fast, but it still felt like ten minutes was needed for us to calm down together.

(Mother 5)

The initial sense of both the parents and nurses was that the preparation time used in the study design was long. The parents felt that ten minutes was a long time to keep singing, but as time went on, they grew into their task and perceived it as natural. The nurses reflected on their sitting and waiting for time to pass, but also noted that they had the time to do it and could see the need for the family to become calmer.

One parent commented on a blood sampling procedure for which her infant did not have the same preparation time. The parent noted that blood sampling without the preparation time required several attempts, and that the infant cried and was not offered a chance to calm down before the venipuncture:

She did not have time to calm down. I needed the preparation to be able to sing and provide skin-to-skin contact in peace. I felt such a huge difference between the venipunctures. I just want to say that the preparation time is really needed, for both me and my infant. I really want to point out that it is needed!

(Mother 1)

#### 3.1.2. Knowledge Affords Agency

The preparations made the parents calm and reduced their stress in relation to the procedure. As part of the preparation, before the procedure started, the nurses informed the parents about their role during the procedure and about parent-delivered pain management. The parents mentioned the need to be ready to take in the given information in order to become active. Some days, the stress of being admitted to the NICU is enough to handle and sensitivity is required from the nurses for the parents not to feel overwhelmed.

Concerns were expressed by both nurses and parents that not all parents would be able to participate in their infant’s pain management due to personal differences. All families are usually given the same information, but the nurses perceived that parents have different abilities to process the information given. Parents felt they had to put their own feelings and stress aside to be there for their infant during the painful procedure.

By gaining knowledge of the benefits of parent-delivered pain management methods, the parents had the opportunity to take an active role. They also felt empowered, as their own previous experiences were confirmed during the focus group. The nurses felt that providing the parents with information allowed the parents to make an active choice to participate in parent-delivered pain management. Communication and information were considered important by both parents and nurses in order to gain knowledge:

I had never heard that kangaroo care actually worked as pain relief. I thought that infants were placed there to keep warm and not much more. But the more you read about it, the more you realize what a bond it creates, since it facilitates development so much.

(Mother 5)

The parents mentioned that both verbal and written information about how they could provide pain management was useful. Sometimes they forgot or did not understand the verbal information, and the possibility to go back and read the written information helped them understand better. This was also emphasized by the nurses, who also believed in a combination of several information media.

#### 3.1.3. Preparation Brings Success

Finding solutions and opportunities to enable combined parent-delivered pain management by generating good conditions and ergonomic adaptations for the infants, parents, and nurses during the procedure was considered important and necessary. The parents mentioned their concern about the nurses’ working conditions, and for example, adjustable parent beds and chairs were used to improve the ergonomic conditions for the nurses. The nurses, however, mentioned that they often overlooked their own ergonomic needs and neglected their own position during procedures. Nonetheless, they were always concerned about the parents’ position and made sure they were comfortable:

I believe that it [i.e., SSC] has become standard treatment, and it feels great to me. We have discussed this earlier, along with position and ergonomic issues. But we have found solutions, so I don’t see any hindrances to doing venipuncture or whatever procedure it is skin to skin. And I don’t get disturbed by someone who hums a little music.

(Nurse 2)

By positioning the infant properly, the procedure was easier for the nurse to perform and also better ensured the wellbeing of the infant. Secure solutions for moving the infant to the parents for SSC were also mentioned as adaptations that had a positive impact on the conditions during procedures.

The nurses felt that their work became easier when the infant was calm and close to the parents during a procedure. They also reflected on the potential barrier posed by inexperienced nurses who feel insecure about their role and need a sense of control during a procedure. All of the nurses expressed a need for clear guidelines in the unit about combined parent-delivered pain management in order for all staff to work toward the same goal:

It is so important to be encouraging and not to be afraid. I believe that if you are uncertain, you think it will be difficult to take blood samples from the infant in the arms of the parent. That if you fail, you believe it would be better to have the infant in a cot or on a changing table because maybe the parents will not be as involved there. But it becomes so much better if you try.

(Nurse 1)

The surroundings in the unit were adapted in different ways to facilitate the procedure. One important adaptation was minimizing sounds, for example, by turning alarms off in the room, during the procedure and preparing the sampling kit outside the room. This was done both for the wellbeing of the infant and to allow the infant to better hear their parents’ voices. The light was also dimmed during the procedure. These adaptations were regularly carried out in the units but received more attention during the research procedure.

Some parents compared the study procedure with previous procedures and recognized that the infant was calmer when combining the methods and giving all parties time to prepare. The nurses performing the procedure also compared the study procedure with other situations and felt a positive difference. The infant’s faster recovery after the procedure was mentioned as an improvement related to the combined parent-delivered pain-alleviating methods.

Parents, nurses, and researchers perceived that all infants were calm and showed no or minor signs of pain during the procedures when performing the combined parent-delivered pain management in this study. This gave a feeling of success and pride for both parents and nurses:

As good as it gets. He did not even notice what happened. He did not react to the needle or anything, so it was totally amazing.

(Mother 2)

### 3.2. Participation

The parents felt that the singing made them shift their attention more toward their infant and that they became more present in the moment. The parents also experienced a feeling of becoming part of a team supporting their infant. All participants described the combined parent-delivered interventions as feasible.

#### 3.2.1. Shifting Attention

The parents’ live lullaby singing enabled them to focus on their infant instead of the procedure. In addition, even parents with needle phobia remained calm, as the singing helped them relax and become more emotionally stable and available to their infant during the procedure. The nurses also observed that the singing calmed the parents, whose attention shifted from what the nurses were doing with the needle toward their infant:

Then I felt calm. I focused on the singing and was not as nervous, and I could also see that he [i.e., my infant] was very calm. I was not as nervous about the venipuncture as I would have been before, because then I would have looked at the needle. But now I did not think about it at all.

(Mother 2)

#### 3.2.2. Parents Support Each Other

The parent couples experienced being a team during their time in the neonatal unit. They said that it was a privilege for both of them to be present and support each other and that they shared a common focus on their infant. The feeling of being a team generated a shared sense of responsibility for their infant’s pain management. In some couples, the parent who felt most comfortable in providing pain management had taken on that role during painful procedures:

It is the most positive thing and especially for me as a father. If he had been born full term, then I would have been off to work now and only seen him parts of the day and you [i.e., the mother] would mostly have been sleeping and breastfeeding.

(Father 5)

Parents mentioned that supporting each other by singing together for the infant during the study procedure was beneficial, making them feel closer and more secure:

I believe, after all that we have gone through, that you provide security for each other as well. It makes it easier when you don’t need to stand alone—we are doing this together.

(Father 2)

#### 3.2.3. Feasible Methods

Both parents and nurses perceived the combined parent-delivered methods used in this study as feasible. Some parents mentioned that singing with their infant on their chest became physically somewhat strenuous after a while, but that it was nevertheless feasible. The parents could not identify any disadvantages of combined parent-delivered pain management. The positive feelings toward combined parent-delivered pain management were due to the feeling that the intervention really helped their infant.

The nurses mentioned limited time as a possible problem, but also stated that they could take the extra time to prepare the family if the venipuncture was not acutely needed. Otherwise, the nurses felt that the combined parent-delivered pain-reducing methods were feasible. One procedure was performed when the infant was admitted to neonatal homecare during a clinical visit. The nurse performing the immunization made extra adaptations, such as placing an adjustable chair in the room. After the study procedure, she decided to keep using this chair to facilitate continuous parent-delivered pain management.

One parent continued to combine SSC and lullaby singing during venipunctures even after the study procedure:

Yes, I have done it [i.e., continue to combine interventions during painful procedures]. One time there were like three staff and one of them was just observing, and I asked her to look at his face. She told me he had not reacted to the venipuncture. So, we continued to do it.

(Mother 3)

### 3.3. Closeness

For the parents, the musical presence generated a feeling of closeness to the infant; together with SSC, this prepared the parents well for combined parent-delivered pain-reducing interventions. The live parental infant-directed lullaby singing created a calm atmosphere around the procedure.

#### 3.3.1. Musical Presence

When the parents used their voices in combination with SSC, they felt that the singing brought them closer to their infant and that they became more responsive to their infant’s signals. The singing also led to a feeling of providing a safe place for their infant, creating a sense of security for both themselves and their infant. The nurses acknowledged the parental voice as a familiar sound for the infant that mediated security and tranquility.

It felt like we came closer and that I was more in the situation. Sometimes we slept with him on us, but now with the humming it was like making some sort of contact. It was something more that helped him and that felt good.

(Mother 4)

The parents reported diverse experiences of singing to their infants both during pregnancy and after delivery. One parent was a musician and had practiced music during pregnancy. Most parents in this study were musically untrained but had an interest in music or had read and heard that singing was beneficial for fetal development. All families practiced lullaby singing during the time between the focus group and the planned study procedure:

You have heard them since you were a child, so they feel cozy. There are so many beautiful lullabies and songs, and it is hard to pick just a few. But there are a few that we remember from our childhood and they mean a lot, a lot of warmth.

(Father 2)

The parents used familiar lullabies that they knew and felt secure with during the procedure. The parents wanted the songs to be easy to repeat and sing for a long time. Some parents sang the same song throughout the procedure and others combined several children’s songs (Appendix B).

#### 3.3.2. Parent–Infant Proximity

The parents felt involved in their infant’s care, which generated a feeling of security for them. By spending long periods of SSC time with their infant, they learned to interpret their infant’s signals. Not having their infant close could in contrast create a feeling of stress and uncertainty about the infant’s condition. They experienced it as instinctive to be close, and being with their infant was the logical position. This proximity also made the combined parent-delivered pain management more natural for the parents. When experiencing the positive effects, they felt encouraged to continue to be active in their infant’s pain management. The parents also expressed that staff encouragement was essential to becoming involved:

We have felt involved, and we talked about those times when we did not feel involved. It felt like our responsibility was taken away from us—it is my child and I want to do everything.

(Mother 5)

Even though some of the parents felt that some painful procedures were hard to witness, they felt that it would be equally difficult not to be present. Their feelings would be the same even if they were not close to their infant, so they chose to stay with the infant to provide support during the procedure.

#### 3.3.3. Calm and Trusting Atmosphere

Both parents and nurses described how the live parental lullaby singing changed the atmosphere in the room. The parents experienced a feeling of harmony, connecting to both each other and their infant. The family’s composed state of mind spread from the parent–infant dyad to the nurses as well, resulting in minimal unnecessary conversations in the room:

From the beginning to the end of the situation, the room was very calm. For me as a parent, the infant, and the staff as well. Because they [i.e., the staff] did not talk to each other. They communicated with gestures—why did they do that? It was so interesting.

(Mother 1)

The nurses also felt calmed by the parents’ singing and said that it reduced their stress as well:

I felt really calm. I think that the singing creates a peaceful atmosphere, and it is impossible to feel stressed in this setting.

(Nurse 3)

## 4. Discussion

The aim of this study was to investigate parents’ and nurses’ perceptions of and reflections on experiencing combined parent-delivered pain management and to explore its application to practice, while the desired outcome of the pain management was to alleviate infant pain during a painful procedure. Based on the analysis of the recorded material and the interpretations of the detailed descriptions, the infants in this study were perceived as calm and showed no or minor signs of pain during their procedure. The infants were observed to have stable vital parameters during and after the procedure, indicating wellbeing throughout the process. Each of the parent-delivered methods studied here has been shown to be pain-alleviating, so by combining several such methods, pain alleviation should be even more efficient [20]. This study employed a unique collaborative design involving the parents as co-researchers in exploring combined parent-delivered pain management together with nurses and researchers.

Based on the analysis of the interviews with parents and nurses, preparation was found to be the key to facilitating combined parent-delivered pain management, in terms of both short- and long-term preparations. The short-term ten-minute preparation time before the procedure was based partly on earlier experience in the present research group [8] and partly on earlier research in which the duration of SSC was varied, with ten minutes being considered the shortest time needed for effective pain relief [7,29]. The preparation time was necessary not only for pain alleviation but also for the parent–infant dyads to connect and calm themselves before the procedure. The non-pharmacological preparations before a painful procedure should consist of a secure environment adapted to the child and where wellbeing and self-efficacy are optimized and maintained [30]. When offering the parent–infant dyad time to calm themselves before a painful procedure, these different aspects of the environment are facilitated, so the self-efficacy of the parent–infant dyad can be better promoted.

The long-term aspect of preparing for combined parent-delivered pain management concerns knowledge. Both nurses and parents stated that information and knowledge were necessary to promote combined parent-delivered pain management. Knowledge facilitates parental agency and empowers the parents to become actively involved in their infant’s pain management [31]. Nurses also need ongoing education, since knowledge will affect their attitudes toward parent-delivered pain management and how they help parents to participate during painful procedures [2,32,33,34,35,36,37]. The nurses’ attitudes and willingness will influence whether the parents are included and able to participate in pain management for their infant. Promoting parent-delivered pain management calls for interprofessional collaboration involving partnership between the healthcare professionals and the parents, with the focus on optimal pain management for the infant [38]. In our research project, collaboration between the parents and nurses was the foundation of the study design within the PAR methodology. The participants became partners in a collaborative exploration of combined parent-delivered pain management. Such partnership should also be the goal in the clinical setting to prevent neonatal pain. In line with family-integrated care (FIC), the parents should receive education and information about their infant’s care in terms of both parent-delivered everyday care for their infant as well as parent-delivered pain management [19]. Different ways of providing information to parents have been investigated, and parents’ needs vary, as emphasized in the present results. Parents also want and need information provided at a time when they can process it, conveyed in a language and using a method they can understand, in a calm setting, and repeatedly throughout their NICU stay [32,33].

When the need for preparation time is met, participation, the second main category, can be achieved, making the combined parent-delivered methods feasible. Even though the nurses and parents found the methods feasible, they could still identify possible barriers to parental-delivered pain management. Such barriers identified in previous research are a restrictive environment, a lack of staff knowledge and training, a lack of time due to workload, everyday life requirements for the parents, and underestimation of the parents [4,32,33,36,39,40]. These barriers are similar to those that the present participants could identify. This indicates that awareness of barriers could lead to adaptations to enable parental presence during painful procedures. Therefore, unit policies, practices, and available guides for utilizing parent-delivered pain management should be adapted to increase the use of non-pharmacological strategies and parental involvement in neonatal pain care [1].

Participation and closeness were the two main categories facilitated by the parents’ live lullaby singing. The singing helped the parents shift their attention and was perceived to increase self-efficacy in the parent–infant dyad. Some parents in this study were afraid of needles, and the singing helped them relax and cope with the situation. This might be explained by increased levels of the stress-protecting hormone oxytocin, as seen in earlier research [41]. Maternal singing during SSC also seems to decrease the mothers’ anxiety [42,43,44]. The singing created a safe, calm, and trusting atmosphere in the procedure room, affecting not only the parent–infant dyad but also the nurses and other staff, including the researchers. The parent’s singing voice is a nurturing resource for hospitalized infants, protecting them from the adverse effects of pain and separation [13]. Early affective and social interactions are important for healthy infant development and could be incorporated in painful procedures using combined parent-delivered pain management, as done in our study, which is the first to investigate the inclusion of live parental lullaby singing in combined parent-delivered pain management.

The importance of parent–infant proximity was expressed as the parents being present, involved, and active in their infant’s care from the beginning. Following the theory of FIC, the parents are considered the primary caregivers of their infant [45]. When the parents are acknowledged as the most important persons in their infant’s life, their role of soothing and comforting their infant during painful procedures is protected and acknowledged. Parental presence in the neonatal units also increases the use of pain management and pain documentation [3,5,46]. In Sweden, FIC is standard in neonatal units, and parental presence is the norm, making the use of parent-delivered pain management feasible. In Sweden and the other Nordic countries, the social insurance systems with generous paid parental leave policies also facilitate the parents’ presence during their infant’s hospitalization. However, not all countries give parents financial support to spend time at the hospital with their hospitalized infant and be present in the unit at all times. Even if the parents cannot be present 24 h a day, non-acute painful procedures could always be planned and performed when the parents are present and can provide pain management for their infant. This was also emphasized by the nurses in this study, who said that their unit historically performed blood sampling during the night shift. Today, the routine has changed, and blood sampling is performed with parental presence and adapted to the infant’s rhythm.

In this study, singing was found to be feasible to use in connection with other parent-delivered pain-reducing methods. The parents in our study also became calmer when singing, because the singing serves as a real-time emotion regulator for both the infants and their singing parents, increasing self-regulation, stabilizing their affect, validating the parental role, and strengthening the mutuality and reciprocity of the parent–infant dyads, as previously shown [47]. More research is needed to develop additional early preventive therapeutic interventions, and live parental lullaby singing as pain management is a new research area in both nursing and music therapy. This study builds on our knowledge of live parental lullaby singing as an underutilized biopsychosocial parent-delivered pain-reducing adjuvant to the combination of SSC and breastfeeding. The efficacy of combined parent-delivered pain management that also includes live parental lullaby singing is currently being tested elsewhere [48].

### Limitations and Strengths

The sample of this study could be considered rather small, but the various data gathering methods used generated rich material capturing diverse aspects of combined parent-delivered pain management. The studied families and nurses were recruited by means of convenience sampling and were interested in pain management. Almost all of the parents had been involved in parent-delivered pain management and SSC before entering this study. This could affect the transferability of the study results, but families always have individual preferences and needs that should be taken into consideration. Nonetheless, the study data were gathered from three independent NICUs in both large and small counties, so the results may provide general guidance on how combined parent-delivered pain management can be perceived and performed in various settings.

Different aspects of analytical triangulation were used in the analysis of this study to avoid bias and achieve trustworthiness [49]. By using various data gathering methods, the consistency of findings could be compared within the varied materials and among the different NICUs. We also used analyst triangulation, in which all of the authors read and interpreted the written material to avoid bias from a single observer.

## 5. Conclusions

Preparation, mental as well as practical, is central to implementing combined parent-delivered pain management. When nurses and parents co-create knowledge, collaboration in the infant’s best interest occurs. Parental preparation for painful procedures creates opportunities to manage participation and is a prerequisite for successful pain management, which prevents and alleviates the infant’s pain. When parents and nurses explored the combined parent-delivered pain-reducing interventions, they found the methods feasible, promoting self-efficacy and confidence in both parents and nurses. The parents felt that the singing brought them closer to their own feelings and thus to their infant, and that their focus in the present moment shifted from the painful procedure to their infant’s wellbeing. The live parental lullaby singing, in combination with SSC and breastfeeding, is considered an accessible resource for both parents and infants, helping them calm down before the procedure and creating a calm and trusting atmosphere during the procedure.

## 6. Application to Practice

Sufficient preparation time, individually assessed and tailored to each parent–infant dyad, is necessary to facilitate parental participation and parent–infant closeness in combined parent-delivered pain management. It is recommended that neonatal units provide support, education programs, and guidelines to enable combined parent-delivered pain management as part of standard non-pharmacological neonatal pain management.

## Figures and Tables

**Table 1:** Demographics of the included parents, their infants, and the nurses.

	*n*	Mean (SD)	%
**Parents** (current and veteran)	11		
Age, years		31 (9.7)	
High school education	2		18
Post-secondary education (not university)	2		18
University education	7		64
**Infants**	5		
Gestational age, weeks + days		28 + 3 (3 + 1)	
Birth weight, grams		1308 (533)	
Postnatal age, weeks + days		34 + 4 (5 + 2)	
Current weight, grams		2638 (710)	
Breathing support			
HFNC	1		20
CPAP	1		20
Ventilator	1		20
**Nurses**	4		
Age, years		34 (6.4)	
Gender, women	4		100
Years worked as a nurse		9.7 (8.3)	
Years worked in neonatal care		9.3 (7.8)	

Note: *n* = number of participants; SD = Standard Deviation; HFNC = high-flow nasal cannula; CPAP = continuous positive airway pressure.

**Table 2:** An overview of the participants in the five focus groups.

	Mother	Father	Veteran Parent	Registered Nurse	Assistant Nurse
Focus group 1	X		X	X	X
Focus group 2	X	X	X	X	X
Focus group 3	X	X	X	X	
Focus group 4	X	X		X	
Focus group 5	X	X		X	

The X in Table 2 marks which participants that participated in the different focus groups. So the X is just to indicate the different participants in the focus groups since all groups did not had the exact same participants.

**Table 3:** The steps of the analysis according to Malterud (2012).

*1. Total impression:* *from chaos to themes*	Reading the entire material to obtain an overall impression of the findings
*2. Identifying and sorting meaning units: from themes to codes*	Meaning units and preliminary codes were identified
*3. Condensation:* *from codes to meanings*	Condensing and extracting the meanings of the preliminary codes into main categories and sub-categories
*4. Synthesizing:* *from condensation to* *descriptions and concepts*	Summarizing the content of each category and describing their meanings

## Data Availability

The data presented in this study are available on request from the corresponding author. The data are not publicly available due to privacy restrictions.

## Appendix A

**Safe, calm, and close to his mother. A detailed description of an immunization procedure with combined parent-delivered pain management in neonatal care.**

Baby boy Oliver is already positioned to have SSC with his mother Sofie—as I will call him and her—when I enter their single-family room. The light is dimmed, and the atmosphere in the room is quiet and calm. The family has stayed together in the family room for a couple of weeks now, and I see a cozy room where the parents have made themselves comfortable. The parents tell me that their boy has been sleeping skin to skin on Sofie’s chest for the last couple of hours. I look at Oliver’s monitor and see a stable pulse and stable oxygen levels. Sofie and Oliver are lying in the parental bed. I bring my video-recording device, and we prepare for the study procedure. Nurse Caroline joins us and sits on a chair next to the family. She has prepared the immunization for Oliver and puts down her tray next to her on a table. She looks calm and is focused on the family.

Baby Oliver stayed three weeks in the open-bay area in the intensive care unit before he was ready to move into the single-family room with his parents. His parents have spent many hours of SSC with him during these weeks. He has started to breastfeed but does not always have enough energy and therefore still needs to be tube feed. So, for the procedure today, the combination of SSC and parental lullaby singing is most suitable due to Oliver’s immaturity. Oliver was fed through his feeding tube half an hour before the procedure and has had some time to process the feeding.

The room is completely calm, but I can also sense a slight tension due to the testing of the combined parent-delivered pain management. Oliver is still sleeping on his mother’s chest. Peter, as I call his dad, sits down next to the bed, as close as he can to his family. We set the timer for ten minutes and the preparation time for the procedure starts. Sofie begins singing a lullaby in a quiet, soft voice. She sings without words, just humming slowly. She and Peter have chosen the song “Baa baa black sheep”, simply just because they felt it was easy to sing. The singing is a bit tentative to begin with, and Sofie looks at Peter and smiles a couple of times. After singing for a minute, Sofie starts to giggle a little and her laugh spreads to all of us, and we all giggle a little before she picks up on her singing again.

After a couple of minutes, baby Oliver makes sounds and starts to move his legs and arms. Sofie shifts his arm a little and changes how she holds him, now holding him gently with both hands over his back and body to support him. It looks secure and supportive, and Oliver seems to like it. He is not completely peaceful and continues to move his legs and make sounds. The sound is not crying, more like a grumbling sound—I would say typical of premature born infants, from my experience as a neonatal nurse working for twelve years.

Oliver is a little frustrated and the alarm of his monitor goes off. Nurse Caroline quiets the alarm and I notice that his pulse is high due to his discontent. I notice that the parents do not seem at all stressed about the alarm. Sofie strokes Oliver’s head and continues to sing in her quiet, soft voice. I am thinking that Oliver might want to change his position, so I tell the parents that it is okay if they need to correct his position. Sofie and Peter tell me that Oliver is upset because he has peed in his diaper—they know him so well after these weeks in the NICU.

Oliver calms down and falls asleep, Sofie is still stroking his head and adjusts his blanket a little. There are a couple of minutes of preparation time left, and Sofie continues to steadily sing the same song. Nurse Caroline gives Oliver oral glucose and offers the pacifier. She looks calm and concentrated. Oliver makes some noise and then, satisfied, starts to suck on his pacifier. Nurse Caroline hands the syringe with oral glucose to Peter. He takes the syringe, and I can tell that he is familiar with the responsibility of administrating glucose. Oliver has previously been through many painful procedures in his first six weeks of life, and his parents are used to supporting him. The ten-minute-long preparation time has passed, and nurse Caroline starts to gently touch Oliver’s left leg to inform him that something is about to happen, and to move his leg into correct position for the immunization. When Caroline wipes Oliver’s leg with disinfectant, he starts to protest a little, but Caroline still looks calm. She is an experienced nurse who has given immunizations many times before. Peter offers Oliver more glucose and he calms down and sucks on the pacifier.

Nurse Caroline gives the injection and after a couple of seconds Oliver starts to cry. My first reaction is that the crying is not that intense and Oliver calms down quickly when Sofie sings in a calm soft voice and embraces him with her hands. Oliver also receives a little bit more glucose from his father, and then calms down after a couple of seconds of sucking intensively on his pacifier. The calm atmosphere in the room returns, and I inform Sofie she can quit singing when Oliver feels calm. She stops singing almost immediately. The parents and nurse Caroline start to chat a little about the procedure. They all seem relaxed and content. I feel a bit of relief in the room as well. I thought this was a successful procedure: Oliver reacted to the immunization, but to me, the reaction seemed milder than how infants normally react to immunizations. He calmed down quickly and had the best possible support during this painful situation. I feel pleased when I look at Oliver, who is now sleeping, safe and calm close to his mother.

## Appendix B

Lullabies used in the intervention:

Bä bä vita lamm (Tegnér A, 1892, Swedish. Translated from Baa baa black sheep, 1744).
Lilla snigel (Trad.: 1957, Swedish).
Björnen sover (Trad.: Swedish).
Sommarsången (Lindgren A and Riedel G, 1970, Swedish).
Videvisan (Tegnér A. and Topelius, Z., 1895, Swedish).
Vem kan segla förutan vind (Samuelsson L, 1971, Swedish).
Kalle Teodor (Lindgren A and Riedel G, 1970, Swedish).
Idas Sommarvisa (Lindgren A and Riedel G, 1973, Swedish).
